# Usefulness of Serial Blood Sampling and PCR Replicates for Treatment Monitoring of Patients with Chronic Chagas Disease

**DOI:** 10.1128/AAC.01191-18

**Published:** 2019-01-29

**Authors:** Rudy Parrado, Juan Carlos Ramirez, Anabelle de la Barra, Cristina Alonso-Vega, Natalia Juiz, Lourdes Ortiz, Daniel Illanes, Faustino Torrico, Joaquim Gascon, Fabiana Alves, Laurence Flevaud, Lineth Garcia, Alejandro G. Schijman, Isabela Ribeiro

**Affiliations:** a Universidad Mayor de San Simón, Cochabamba, Bolivia; b Instituto de Investigaciones en Ingeniería Genética y Biología Molecular Dr. Héctor N. Torres (INGEBI-CONICET), Buenos Aires, Argentina; c Drugs for Neglected Diseases initiative (DND*i*), Geneva, Switzerland; d Universidad Autónoma Juan Misael Saracho, Tarija, Bolivia; e Fundación CEADES, Cochabamba, Bolivia; f Barcelona Centre for International Health Research (CRESIB), Barcelona, Spain; g Médicins Sans Frontières (MSF), Geneva, Switzerland

**Keywords:** Chagas disease, PCR, *Trypanosoma cruzi*, benznidazole, clinical trials, ravuconazole, treatment monitoring

## Abstract

This work evaluated a serial blood sampling procedure to enhance the sensitivity of duplex real-time quantitative PCR (qPCR) for baseline detection and quantification of parasitic loads and posttreatment identification of failure in the context of clinical trials for treatment of chronic Chagas disease, namely, DNDi-CH-E1224-001 (ClinicalTrials.gov registration no. NCT01489228) and the MSF-DNDi PCR Sampling Optimization Study (NCT01678599). Patients from Cochabamba (*n* = 294), Tarija (*n* = 257), and Aiquile (*n* = 220) were enrolled. Three serial blood samples were collected at each time point, and qPCR triplicates were tested for each sample. The first two samples were collected during the same day and the third one 7 days later. A patient was considered PCR positive if at least one qPCR replicate was detectable. Cumulative results of multiple samples and qPCR replicates enhanced the proportion of pretreatment sample positivity from 54.8% to 76.2%, 59.5% to 77.8%, and 73.5% to 90.2% in Cochabamba, Tarija, and Aiquile cohorts, respectively. This strategy increased the detection of treatment failure from 72.9% to 91.7%, 77.8% to 88.9%, and 42.9% to 69.1% for E1224 low-, short-, and high-dosage regimens, respectively, and from 4.6% to 15.9% and 9.5% to 32.1% for the benznidazole arm in the DNDi-CH-E1224-001 and MSF-DNDi studies, respectively. The addition of the third blood sample and third qPCR replicate in patients with nondetectable PCR results in the first two samples gave a small, non-statistically significant improvement in qPCR positivity. No change in clinical sensitivity was seen with a blood volume increase from 5 to 10 ml. The monitoring of patients treated with placebo in the DNDi-CH-E1224-001 trial revealed fluctuations in parasitic loads and occasionally nondetectable results. In conclusion, a serial sampling strategy enhanced PCR sensitivity to detecting treatment failure during follow-up and has the potential for improving recruitment capacity in Chagas disease trials, which require an initial positive qPCR result for patient admission.

## Abstract (precis)

This work evaluated a serial blood sampling procedure to enhance the sensitivity of duplex real-time quantitative PCR (qPCR) for baseline detection and quantification of parasitic loads and posttreatment identification of failure in the context of clinical trials for treatment of chronic Chagas disease, namely, DNDi-CH-E1224-001 (ClinicalTrials.gov registration no. NCT01489228) and the MSF-DNDi PCR Sampling Optimization Study (NCT01678599).

## INTRODUCTION

Following years of little progress in research and development of new compounds for treatment of Chagas disease (CD), new chemical classes and alternative treatment regimens have demonstrated encouraging activity against its causative agent, Trypanosoma cruzi (1–4). The efficacy of anti-T. cruzi compounds has habitually been measured by means of parasite detection or antibody titers. However, in chronically infected patients, traditional parasitological methods lack sensitivity and T. cruzi-specific antibody titers usually do not decrease until many years after treatment (5). In this context, molecular methods, such as conventional and real-time quantitative PCR (qPCR) assays, have opened promising opportunities for monitoring bloodstream parasitic levels to detect therapeutic failure or response (6–9). Following this approach, multicenter PCR studies have allowed harmonization and validation of standard operating procedures (SOPs) for PCR-based detection and quantification of T. cruzi DNA in blood samples (10, 11) coupled with external control quality assurance (12). However, the best-performing qPCR methods reached between 60% and 70% positivity in untreated chronic Chagas disease patients when a single baseline blood sample was tested (10, 11, 13), a figure which has been verified in different clinical trials (14–16).

In clinical trials in which eligibility criteria for patient enrollment includes PCR positivity, such low values of sensitivity require that a larger proportion of seropositive subjects must be screened before being admitted. To overcome this limitation, a PCR Sampling Optimization Study (ClinicalTrials.gov registration no. NCT01678599) was developed by the Drugs for Neglected Diseases Initiative (DNDi) and Médecins Sans Frontières (MSF). Their aim was to evaluate sampling conditions for qPCR monitoring of benznidazole (BZN) treatment. DNDi-CH-E1224-001, a DNDi-sponsored randomized clinical trial (NCT01489228) to evaluate safety and efficacy of three oral regimens of E1224 (ravuconazole prodrug) compared with those of BZN and placebo, planned to collect three serial peripheral blood samples from each patient at each follow-up time point and perform qPCR in triplicate from each blood sample DNA extract.

This report presents the data obtained in these studies, showing an improvement in qPCR clinical sensitivity for both enrollment and detection of treatment failure in adult patients with chronic Chagas disease.

## RESULTS

### Screening of pretreated chronic CD patients in DNDi-CH-E1224-001 and MSF-DNDi PCR sampling optimization studies.

**FIG 1:**
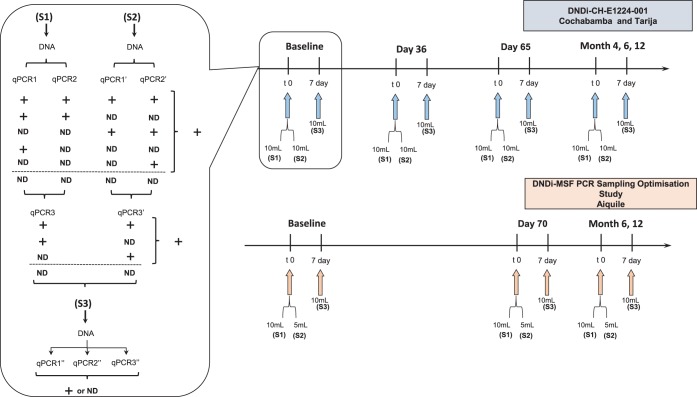
Study diagram and schedule of qPCR assessments.

**(i) Analysis of qPCR replicates in the DNDi-CH-E1224-001 trial.** In the DNDi trial, qPCR was first performed in duplicate from each S1 and S2 DNA extracts. When both replicates gave nondetectable qPCR results from one of these DNA extracts, a third qPCR replicate was analyzed from the corresponding sample. When the third replicate was included, qPCR positivity increased from 54.8% to 60.5% (S1) and from 53.6% to 59.2% (S2) in samples collected from the Cochabamba cohort and from 59.5% to 63.4% (S1) and from 55.3% to 60.7% (S2) in those collected from the Tarija cohort (*P* > 0.05) (Table 1 and Fig. 1).

**(ii) Analysis of serial blood samples.** In the DNDi-CH-E1224-001 trial, the comparison of qPCR positivity obtained after testing individual S1 or S2 samples did not give significant differences (*P* > 0.05) (Table 1), but qPCR positivity increased when cumulative results from the combined S1 and S2 samples (termed S1+S2) were computed; this was observed in both Cochabamba (60.5 versus 69.7%; *P* < 0.05) and Tarija cohorts (63.4 versus 73.9%; *P* < 0.05).

**TABLE 1:** Accumulative qPCR findings in pretreated chronic Chagas disease patients from DNDi-CH-E1224-001 and MSF-DNDi PCR sampling optimization clinical studies^*a*^

Clinical trial, locality, and parameter	Value(s) for:						
	S1		S2		S1+S2	S3	S1+S2+S3
	qPCR1+2	qPCR1+2+3	qPCR1+2	qPCR1+2+3		qPCR1+2+3	
DNDi-CH-E1224-001							
CBBA							
*N*	294	294	289	289	294	74	294
No. (%) positive	161 (54.8)	178 (60.5)	155 (53.6)	171 (59.2)	205 (69.7)	19 (25.7)	224 (76.2)
No. (%) quantifiable	31 (19.3)	31 (17.4)	26 (16.8)	26 (15.2)	44 (21.5)	0 (0.0)	44 (19.6)
Median (IQR) (par. eq./ml)	2.6 (1.9–3.4)	2.6 (1.9–3.4)	2.7 (2.0–3.9)	2.7 (2.0–3.9)	2.6 (2.0–3.5)		2.6 (2.0–3.5)
Tarija							
*N*	257	257	257	257	257	53	257
No. (%) positive	153 (59.5)	163 (63.4)	142 (55.3)	156 (60.7)	190 (73.9)	10 (18.9)	200 (77.8)
No. (%) quantifiable	37 (24.2)	37 (22.7)	32 (22.5)	33 (21.2)	49 (25.8)	0 (0.0)	49 (24.5)
Median (IQR) (par. eq./ml)	2.4 (2.0–3.4)	2.4 (2.0–3.4)	3.0 (2.4–3.6)	3.0 (2.2–3.6)	2.6 (2.0–3.6)		2.6 (2.0–3.6)
MSF–DNDi sampling study							
Aiquile							
*N*		196		195	201	176	205
No. (%) positive		144 (73.5)		150 (76.9)	171 (85.1)	128 (72.7)	185 (90.2)
No. (%) quantifiable		34 (23.6)		40 (26.7)	51 (29.8)	29 (22.7)	61 (33.0)
Median (IQR) (par. eq./ml)		2.4 (1.9–4.5)		2.9 (1.9–4.9)	2.8 (1.9–4.8)	3.2 (2.0–4.8)	3.0 (2.0–4.7)

a S1-3, samples 1 to 3; qPCR1-3, qPCR replicates 1 to 3; CBBA, Cochabamba; *N*, number of samples.

When S1 and S2 gave nondetectable qPCR results, a third sample (S3) was taken 7 days later. The analysis of PCR positivity obtained using three serial samples (S1+S2+S3) compared to that obtained from individual samples demonstrated higher sensitivity for both Cochabamba (60.5 versus 76.2%; *p* < 0.001) and Tarija cohorts (63.4 versus 77.8%; *p* < 0.001). Finally, qPCR positivity obtained after testing S1+S2 versus that obtained after testing S1+S2+S3 increased by 6.5% (*n* = 19/294) in Cochabamba and 3.9% (*n* = 10/257) in Tarija cohorts (Table 1) (*P* > 0.05).

On the other hand, no statistical difference was observed in qPCR positivity by testing individual S1, S2, or S3 samples in the MSF-DNDi PCR Sampling Optimization Study (*P* > 0.05) (Table 1). Computing the cumulative qPCR positivity obtained for S1+S2 (85.1%) compared to the positivity obtained for S1 (10 ml of blood, 73.5%; *P* < 0.01) or S2 (5 ml of blood, 76.9%; *P* < 0.05) alone increased sensitivity. This was also true for the cumulative qPCR positivity obtained for S1+S2+S3 (90.2%) compared to that obtained for the individual samples (S1, *P* < 0.001; S2, *P* < 0.001; and S3, 72.7%, *P* < 0.001). Comparison of the cumulative qPCR positivity obtained from S1+S2+S3 with respect to S1+S2 showed an increase of 5.1% (Table 1) (*P* > 0.05).

**(iii) Analysis of T. cruzi discrete typing units (DTUs) and parasitic loads.** It is worth noting the higher qPCR positivity obtained in patients from Aiquile (90.2%) than from patients recruited from Cochabamba (76.2%; *P* < 0.001) and Tarija (77.8%; *P* < 0.001), whereas no difference was found between the two E1224 trial cohorts (Table 1) (*P* > 0.05). Because both studies used the same qPCR method performed in the same laboratory, a hypothesis for this geographical variability in qPCR positivity could be related to diversity of T. cruzi strains or parasitic loads in the populations studied and/or to a higher endemicity and exposure to the vector in Aiquile and, therefore, a potential risk of reinfection. In order to investigate this, the distribution of T. cruzi DTUs was analyzed by genotyping the 180 qPCR-positive samples from these localities with the highest parasitic loads.

The diversity of the T. cruzi genome and the multiplicity of its genotypes and phenotypes are well recognized. Currently, T. cruzi is partitioned into seven DTUs: TcI to TcVI and Tcbat. In this study, DTUs could be identified in 31 samples: 23 patients were infected with parasite populations belonging to the group TcII/V/VI, six patients were infected with TcI, and two presented mixed infections by TcI plus TcII/V/VI (Table 2). TcI was five times more frequent in Cochabamba and Tarija than in Aiquile, although the small number of genotyped samples preclude determination of its significance. TcIII and TcIV were not detected.

**TABLE 2:** Direct identification of T. cruzi DTUs in blood samples of pretreated chronic Chagas disease patients from DNDi-CH-E1224-001 and MSF-DNDi PCR sampling optimization clinical studies

Clinical trial	Locality	T. cruzi discrete typing unit			
		TcI	TcI+II/V/VI	TcII/V/VI	TcV/VI
DNDi-CH-E1224-001	CBBA-Tarija	5	1	10	1
MSF-DNDi Sampling study	Aiquile	1	1	11	1

**FIG 2:**
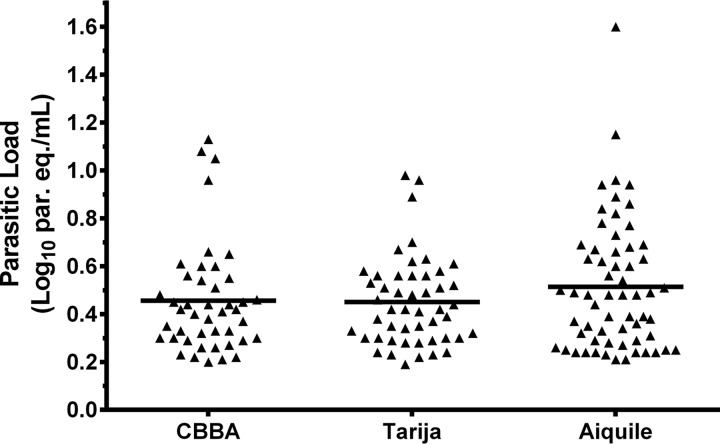
Distribution of parasitic loads in peripheral blood samples of pretreated chronic Chagas disease patients from DNDi-CH-E1224-001 and MSF-DNDi PCR sampling optimization clinical studies. CBBA, Cochabamba.

The parasitic loads of baseline samples from the three different cohorts are shown in Fig. 2. In Aiquile, 33.0% of samples had parasitic loads above the qPCR limit of quantification (LOQ) of 1.53 parasite equivalents in 1 ml of blood (par. eq./ml), whereas in Cochabamba and Tarija the percentage of quantifiable samples was 19.6% and 24.5%, respectively (Table 1). The median and interquartile range (IQR) values of the quantifiable parasitic loads were 2.6 (2.0 to 3.5), 2.6 (2.0 to 3.6), and 3.0 (2.0 to 4.7) par. eq./ml for Cochabamba, Tarija, and Aiquile cohorts, respectively (*P* > 0.05) (Table 1).

### Follow-up of treated chronic CD patients in DNDi-CH-E1224-001 and MSF-DNDi PCR sampling optimization studies

**(i) Analysis of qPCR positivity and parasitic loads.** Table 3 shows the cumulative qPCR findings obtained from all three serial blood samples during screening and monitoring of all treatment arms in both clinical trials.

**TABLE 3:** qPCR findings during baseline and followup of the different groups of treatment of DNDi-CH-E1224-001 and MSF-DNDi PCR sampling optimization clinical studies^*a*^

Clinical trial, treatment group, and parameter	Value(s) (S1+S2+S3) at:				
	BL	2 mo	4 mo	6 mo	12 mo
DNDi-CH-E1224-001					
Placebo					
*N*	46	46	46	46	46
No. (%) positive	46 (100)	34 (73.9)	37 (80.4)	40 (87.0)	36 (78.3)
No. (%) quantifiable	9 (19.6)	15 (44.1)	9 (24.3)	14 (35.0)	16 (44.4)
Median (IQR) (par. eq./ml)	2.2 (2.0-4.1)	2.2 (1.9–4.3)	3.3 (2.1–4.1)	3.1 (2.1–3.7)	2.7 (1.9–5.3)
E1224 LD					
*N*	48	48	48	48	47
No. (%) positive	48 (100)	5 (10.4)	18 (37.5)	32 (66.7)	36 (76.6)
No. (%) quantifiable	14 (29.2)	0 (0.0)	6 (33.3)	10 (31.3)	12 (33.3)
Median (IQR) (par. eq./ml)	3.5 (2.6–7.0)		2.1 (1.9–2.5)	2.1 (1.7–2.4)	2.2 (2.1–4.3)
E1224 SD					
*N*	45	45	44	43	45
No. (%) positive	45 (100)	4 (8.9)	31 (70.5)	33 (76.7)	38 (84.4)
No. (%) quantifiable	9 (20.0)	0 (0.0)	8 (25.8)	5 (15.2)	12 (31.6)
Median (IQR) (par. eq./ml)	2.3 (2.0–2.9)		2.5 (1.9–3.5)	2.2 (1.9–2.6)	2.8 (2.1–4.5)
E1224 HD					
*N*	42	42	41	41	41
No. (%) positive	42 (100)	7 (16.7)	9 (22.0)	14 (34.1)	23 (56.1)
No. (%) quantifiable	11 (26.2)	0 (0.0)	0 (0.0)	1 (7.1)	6 (26.1)
Median (IQR) (par. eq./ml)	2.5 (1.9–3.4)			1.8	2.0 (1.9–2.2)
BZN					
*N*	44	44	43	43	44
No. (%) positive	44 (100)	3 (6.8)	0 (0.0)	2 (4.7)	2 (4.5)
No. (%) quantifiable	11 (25.0)	0 (0.0)	0 (0.0)	0 (0.0)	0 (0.0)
Median (IQR) (par. eq./ml)	2.1 (1.9–2.7)				
MSF-DNDi sampling study					
BZN					
*N*	137	121		115	116
No. (%) positive	137 (100)	28 (23.1)		11 (9.6)	6 (5.2)
No. (%) quantifiable	47 (34.3)	0 (0.0)		1 (9.1)	0 (0.0)
Median (IQR) (par. eq./ml)	2.8 (1.9–4.6)			2.2	

a BL, baseline. LD, SD, and HD, low, short, and high dosages.

The qPCR positivity of the placebo group from the DNDi-CH-E1224-001 clinical trial was significantly higher at baseline (100%, per study entry criteria) than at the follow-up time points (2 months, 73.9%, *P* < 0.01; 4 months, 80.4%, *P* < 0.01; 6 months, 87.0%, *P* < 0.05; 12 months, 78.3%, *P* < 0.01), whereas no differences were found between follow-up time points (Table 3) (*P* > 0.05). Out of the patients who received placebo, 27 were persistently qPCR positive, 15 had intermittently positive and nondetectable results, and four became persistently qPCR undetectable during follow-up.

In both clinical trials, the treated cohorts showed a drastic reduction in PCR positivity at the end of treatment (EOT) (E1224 low dose [LD; 8 weeks], 10.4%; E1224 short dose [SD; 4 weeks], 8.9%; E1224 high dose [HD; 8 weeks], 16.7%; DNDi-CH-E1224-001, BZN, 6.8%; DNDi-MSF Sampling Optimization Study, BZN, 23.1%) (Table 3) (*P* < 0.001)[In the abstract and here at its first use in the text, please define “short dose”]. In the E1224 treatment arms, qPCR positivity increased during posttreatment follow-up, reaching its highest value at the end of the study (E1224 LD, 76.6%, *P* < 0.001; E1224 SD, 84.4%, *P* < 0.001; E1224 HD, 56.1%, *P* < 0.01), whereas in the cohorts treated with BZN, the proportion of qPCR-positive cases diminished at the end of follow-up (DNDi-CH-E1224-001, BZN, 4.5%, *P* > 0.05; DNDi-MSF Sampling Optimization Study, BZN, 5.2%, *P* > 0.01).

Interestingly, all treatment arms showed statistically significant differences between the proportion of positive qPCR results at baseline and the end of follow-up (E1224 LD, *P* < 0.01; E1224 SD, *P* < 0.05; E1224 HD, *P* < 0.001; DNDi-CH-E1224-001, BZN, *P* < 0.001; DNDi-MSF Sampling Optimization Study, BZN, *P* < 0.001).

**FIG 3:**
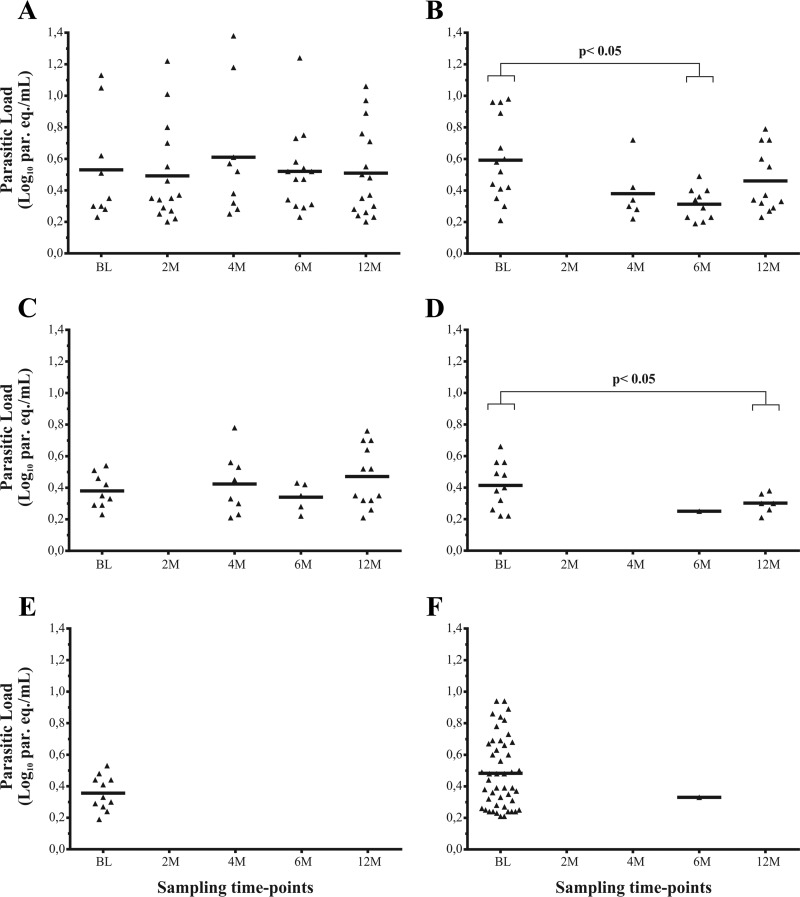
Distribution of parasitic loads during baseline and follow-up of the different groups of treatment of DNDi-CH-E1224-001 and MSF-DNDi PCR sampling optimization clinical studies. (A) E1224, placebo arm; (B) E1224, low-dose arm; (C) E1224, short-dose arm; (D) E1224, high-dose arm; (E) benznidazole arm from the DNDi-CH-E1224-001 trial; (F) benznidazole arm from the MSF-DNDi PCR Sampling Optimization Study. BL, baseline; 2 M, 4 M, 6 M, and 12 M, 2, 4, 6, and 12 months from the beginning of the study.

The number of patients with quantifiable qPCR results from the placebo group of the E1224 trial ranged between 14 and 16 during follow-up, except at 4 months, when, as at baseline, nine patients rendered quantifiable qPCR results (Table 3). Out of the nine patients enrolled in the placebo group of DNDi-CH-E1224-001 who showed quantifiable parasitic loads at baseline, five had quantifiable results throughout follow-up, two patients alternated between quantifiable and nonquantifiable results, and the two remaining showed persistent detectable but nonquantifiable qPCR results throughout follow-up. No significant differences were found among the medians of parasitic loads at baseline and follow-up time points in the placebo group of the DNDi-CH-E1224-001 trial (Fig. 3A) (*P* > 0.05).

Patients treated with E1224 showed nonquantifiable parasitic loads at the end of treatment, but this increased later on; indeed, 12 cases reached quantifiable loads for E1224 LD and SD regimens and six in the E1224 HD regimen at the end of follow-up, whereas in BZN-treated groups only one patient had parasitic loads higher than 1.53 par. eq./ml during follow-up (Table 3). Statistically significant differences were observed between parasitic loads at baseline and after 6 months for E1224 LD (3.5 [2.6 to 7.0] and 2.1 [1.7 to 2.4] par. eq./ml, respectively; *P* < 0.05) (Fig. 3B) and between baseline and 12 months (2.5 [1.9 to 3.4] and 2.0 [1.9 to 2.2] par. eq./ml; *P* < 0.05) for E1224 HD (Fig. 3D).

**FIG 4:**
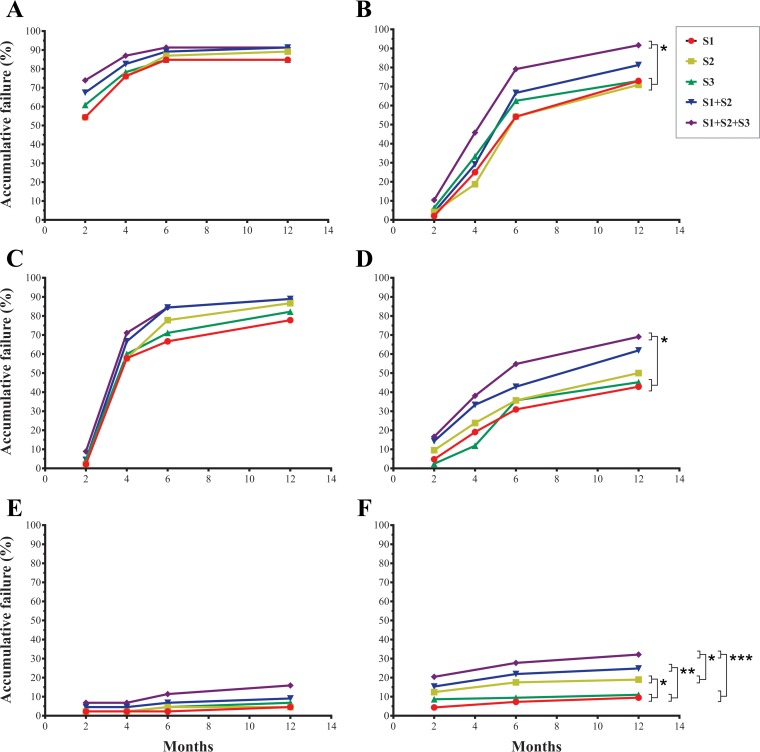
Cumulative therapeutic failure during the follow-up of the different treatment groups of DNDi-CH-E1224-001 and MSF-DNDi PCR sampling optimization clinical studies. (A) E1224, placebo arm; (B) E1224, low-dose arm; (C) E1224, short-dose arm; (D) E1224, high-dose arm; (E) benznidazole arm from the DNDi-CH-E1224-001 trial; (F) benznidazole arm from the MSF-DNDi PCR Sampling Optimization Study. S1 to S3, samples 1 to 3. *, *P* < 0.05; **, *P* < 0.01; ***, *P* < 0.001.

**(ii) Analysis of cumulative therapeutic failure.** Figure 4 analyzes the cumulative qPCR positivity as a measure of treatment failure obtained for each treatment group in both clinical trials from EOT until the end of follow-up.

In the DNDi-CH-E1224-001 trial, the multisampling strategy (S1+S2+S3) increased detection of treatment failure at the end of follow-up by up to 91.7% for E1224 LD (*P* < 0.05) (Fig. 4B), 88.9% for E1224 SD (*P* > 0.05) (Fig. 4C), 69.1% for E1224 HD (*P* < 0.05) (Fig. 4D), and 15.9% for BZN (*P* > 0.05) (Fig. 4E) arms. No significant differences were found between the cumulative treatment failure detected for single S1 (72.9, 77.8, 42.9, and 4.6%), S2 (70.8, 86.7, 50.0, and 4.6%), and S3 (72.9, 82.2, 45.2, and 6.8%) samples and comparing S1+S2 (81.3, 88.9, 61.9, and 9.1%) versus S1+S2+S3 for E1224 LD, SD, and HD, and BZN arms, respectively (Fig. 4) (*P* > 0.05).

In the MSF-DNDi PCR Sampling Optimization Study, the strategy involving serial sampling analysis allowed an increase in detection of treatment failure of up to 32.1% (S1+S2+S3) at the end of follow-up compared to that detected from individual samples (S1, 9.5%, *P* < 0.001; S2, 19.0%, *P* < 0.05; S3, 11.0%, *P* < 0.001). Significant difference was found between the cumulative treatment failure of S1 and S2 (*P* < 0.05), whereas no differences were found between S3 and S1 or S2 (*P* > 0.05) (Fig. 4F). There was an increase of 7.3% in cumulative treatment failure detected after testing S1+S2+S3 versus that detected after testing S1+S2 (24.8%) (*P* > 0.05) (Fig. 4F).

Analysis of cumulative therapeutic failure among the different treatment groups of the E1224 trial did not show significant differences among placebo and E1224 LD and SD arms (*P* > 0.05) (Fig. 4). In contrast, the E1224 HD arm showed lower treatment failure than placebo (*P* < 0.05) and E1224 LD (*P* < 0.01) and SD (*P* < 0.05) groups. In addition, the DNDi-CH-E1224-001 BZN group showed lower treatment failure than placebo and E1224 arms (*P* < 0.001).

No statistically significant differences were observed between the cumulative therapeutic failure of BZN-treated cohorts enrolled in DNDi-CH-E1224-001 and MSF-DNDi PCR sampling optimization studies (Fig. 4) (*P* > 0.05).

## DISCUSSION

### Impact of serial sampling strategies on qPCR sensitivity.

In recent years, several clinical trials to evaluate antiparasitic treatments for CD were carried out using different sampling strategies and PCR protocols, and various rates of PCR positivity were obtained (14, 15, 17).

The present analyses show that qPCR sensitivity was significantly improved at baseline in the DNDi-CH-E1224-001 trial when two blood samples were collected and each DNA extract was analyzed in duplicate by qPCR. The addition of the third blood sample and third qPCR replicate in the subset of patients who had nondetectable PCR results for S1 and S2 gave a small but non-statistically significant improvement in positivity. The limited data available thus far are insufficient to determine the clinical relevance of this small increase in qPCR sensitivity in the evaluation of treatment response. In fact, the samples with only one out of three PCR-positive results were nonquantifiable. As treatment was expected to reduce further the parasite burden in those patients with nonquantifiable baseline qPCR results, reducing the chance of detecting treatment failure, three blood samples and qPCR triplicates were tested during posttreatment follow-up.

In the MSF-DNDi PCR Sampling Optimization Study, the use of 5 ml of blood instead of 10 ml as the starting sample for qPCR analysis, as well as the collection of a third blood sample 7 days after the first two samples instead of few minutes later, did not modify the overall clinical sensitivity.

In conclusion, these findings support the use of lower volumes of blood, collected during the same visit, for qPCR testing purposes.

### Distribution of DTUs and parasitic loads.

TcV was the prevailing DTU, in agreement with findings reported by Martinez-Perez et al. (18), who found TcV in 55.2% of Bolivian CD patients living in Madrid, Spain. However, TcIV, usually associated with the sylvatic cycle and occasional oral outbreaks (19, 20) but found as the second predominant DTU in Bolivian patients (18), was not detected.

Differences in qPCR positivity between Cochabamba or Tarija cohorts and the Aiquile cohort could be attributed to different distributions of parasite DTUs in these localities, such as was observed for TcI (Table 2), although the small number of genotyped samples precluded assessment of the significance of this finding.

Median parasitic loads were higher in Aiquile than in Cochabamba or Tarija, although the differences did not reach statistical significance (Table 1 and Fig. 1). This could be due to the rural nature of the Aiquile area compared to the cities of Cochabamba and Tarija. In a recent study of pregnant women from Bolivia, it was observed that the differences in seroprevalence for T. cruzi infection were, above all, related to the area in which the patients lived most of their lives. Hotspots where disease is hyperendemic were observed where prevalence surpassed 60%, and one of the affected areas was the municipality of Aiquile, with 66% seroprevalence (21). In areas where vector infestation was higher, the seroprevalence of CD was also higher (21).

### Dynamics of bloodstream parasite burden in chronic CD.

The monitoring of samples from patients treated with placebo in the DNDi-CH-E1224-001 trial allowed follow-up of the natural history of human chronic T. cruzi infection in adult patients for a period of 1 year. The results showed that a proportion of patients had fluctuations of parasitic loads, which, in some cases, fell below the LOQ (1.53 par. eq./ml) of the qPCR method (13) and even gave nondetectable results, reflecting the fluctuations of parasitemia observed in chronic CD patients using traditional parasitological methods (20). Such findings underscore the need for serial sampling and qPCR replicate analysis for the evaluation of therapeutic failure in chronic CD.

### qPCR as surrogate marker of therapeutic failure in CD clinical trials.

The qPCR-based study of the DNDi-CH-E1224-001 clinical trial demonstrated that BZN was a better parasiticidal drug than E1224 in monotherapy and that, in turn, E1224 HD had higher efficacy than the other E1224 regimens (Fig. 3). Treatment with BZN gave a better parasitological response in the urban cohorts of the DNDi-CH-E1224-001 trial than in the rural patients from the MSF-DNDi PCR sampling study, although no significant differences were found. This could be due to the more controlled conditions of treatment administration and follow-up in the DNDi-CH-E1224-001 trial rather than to a higher risk of reinfection in the rural community of Aiquile, since the houses of all patients enrolled in the MSF-DNDi PCR sampling study were under entomological surveillance.

Finally, this report demonstrates the usefulness of serial blood sampling and qPCR replicate analysis not only for enhancing the capacity to recruit chronic CD adult patients for clinical trials, in which the inclusion criteria require at least one qPCR-positive result at baseline, but also, more importantly, for increasing sensitivity to detect treatment failure in this population. At the same time, this work highlights the importance of standardized methods for monitoring treatment response in chronic CD.

## MATERIALS AND METHODS

### Ethics statement.

The clinical trials, including the sampling requirements, were approved by the Ethical Review Boards of Universidad Mayor de San Simón, Fundación CEADES, Hospital Clínic, and Médecins Sans Frontières by following the principles expressed in the Declaration of Helsinki. Written informed consent forms were signed by the study volunteers (no minor subjects were included in these trials). All samples were anonymized before being processed.

### Subjects and samples.

Subjects were recruited for two different clinical studies.

**(i) DNDi-CH-E1224-001.** The DNDi-CH-E1224-001 clinical trial (NCT01489228), designed and sponsored by DNDi, with a proof-of-concept double-blinded randomized design aiming to evaluate the safety and efficacy of three (high-, low-, and short-dose) oral regimens of E1224, compared to BZN (5 mg/kg of body weight/day) and placebo treatment, during 60 days of treatment of adult patients with chronic indeterminate Chagas disease (22). A total of 560 patients aged 18 to 50 years and serologically confirmed as having Chagas disease were screened in two study sites of The Platform for a Comprehensive Care of Patients with Chagas Disease in Bolivia, one site in the city of Cochabamba and the other in the city of Tarija. Of those screened, 551 patients had PCR results available for analyses, as a total of 9 patients withdrew consent for participation and no PCR sample was collected.

Samples consisted of peripheral blood mixed with an equal volume of 6 M guanidine hydrochloride, 0.2 M EDTA, pH 8.0, buffer. A maximum of three 10-ml blood samples were collected at baseline: sample 1 (S1) and sample 2 (S2) were collected on the same day and sample 3 (S3) was collected 7 days later, but only if DNA extracts from S1 and S2 gave nondetectable results (as depicted in Fig. 1). The qPCR was performed in duplicate from both S1 and S2 DNA extracts. In cases where both replicates gave nondetectable results, a third replicate was analyzed. When all qPCR replicates from both S1 and S2 gave nondetectable results, S3 was collected and assayed in triplicate. During follow-up, three blood samples were collected at each time point (EOT and 2, 4, and 10 months posttreatment), and qPCR was performed in triplicate from each S1, S2, and S3 DNA extract (Fig. 1).

**(ii) PCR Sampling Optimization Study.** The PCR Sampling Optimization Study (NCT01678599), launched by DNDi and MSF, aimed to evaluate sampling strategies for qPCR treatment monitoring in adult patients with chronic Chagas disease (with indeterminate or early target organ involvement) treated with BZN (5 mg/kg/day) for 60 days. This study was carried out in 17 communities in the rural locality of Aiquile and did not include a placebo or other comparison treatment group. A total of 220 patients aged 18 to 60 years with serologically confirmed Chagas disease were recruited for this trial, but only those with qPCR results at baseline were considered in this work (*n* = 205). All houses of patients entering the study were subjected to entomological surveillance.

From each seropositive patient, three blood samples were collected at baseline and at each follow-up visit (EOT and 4 and 10 months posttreatment) (Fig. 1). S1 and S2 were collected on the same day and S3 7 days later. S1 and S3 consisted of 10 ml of blood, whereas for S2 5 ml was collected; all samples were mixed with an equal volume of guanidine-EDTA buffer. qPCR was performed in triplicate from each S1, S2, and S3 DNA extract (Fig. 1).

Only patients with at least one positive result out of a maximum of nine qPCR replicates were enrolled in these trials. In both studies, therapeutic failure was defined as the persistence of parasite DNA, detected in at least one qPCR replicate, at any time point during posttreatment follow-up.

### DNA extraction.

The High Pure PCR template preparation kit (Roche Diagnostics Corp., Indianapolis, IN) was used to process 300 μl of each guanidine-EDTA-blood (GEB) sample, and DNA was eluted in 100 μl elution buffer, as previously described (13).

### Quantitative real-time PCR procedure.

A duplex qPCR targeted to T. cruzi satellite DNA (SatDNA) and an internal amplification control (IAC) were used as previously described (13). The qPCR reactions were carried out with the use of FastStart universal probe master mix (Roche Diagnostics GmbHCorp., Mannheim, Germany) with 5 μl DNA extract in a final volume of 20 μl. Cycling conditions were a first step of 10 min at 95°C, 40 cycles at 95°C for 15 s, and a final step of 1 min at 58°C. The amplifications were carried out in a Rotor-Gene Q (Corbett LifeScience, Cambridgeshire, United Kingdom) real-time PCR device.

For quantification purposes, standard curves were plotted with 1/10 serial dilutions of total DNA obtained from a GEB seronegative sample spiked with 10^5^ par. eq./ml LL014-1-R1 Cl1 T. cruzi stock (TcV) cultured epimastigotes. One negative control and two positive controls containing 10 and 1 fg/μl T. cruzi CL-Brener DNA were included in every run, as recommended (23).

### Genotyping of T. cruzi discrete typing units.

Baseline samples from both clinical studies with SatDNA qPCR *C_T_* (threshold cycle) values below 33 (*n* = 180) were genotyped using PCR-based strategies targeted to nuclear genomic markers, namely, (i) spliced leader intergenic region (SL-IR)-based PCR was used to distinguish TcI (150 bp), TcII, TcV, and TcVI (157 bp) from TcIII and TcIV (200 bp); (ii) heminested SL-IR-I PCR was used to confirm TcI (350 bp), and heminested SL-IR-II PCR was used to confirm TcII, TcV, and TcVI (300 bp); (iii) heminested PCR of the 24S alpha-ribosomal DNA (24Sα-rDNA) was used to distinguish TcV (125 bp) from TcII and TcVI (140 bp); and (iv) heminested PCR targeted to genomic fragment A10 was used to discriminate TcII (580 bp) from TcVI (525 bp) (24).

Samples that yielded positive results by SL-IR-II PCR but were nondetectable by 24Sα-rDNA PCR were reported as belonging to the TcII/V/VI group. Those samples that amplified the 140 bp of 24Sα-rDNA fragment but had nondetectable results for A10 fragment-based PCR were reported as belonging to the TcII/VI group. Those samples amplifying both bands of 125 and 140 bp after 24Sα-rDNA PCR were interpreted as mixed infections by TcV plus TcII and/or TcVI, as previously described (24).

### Statistical analysis.

McNemar's test was used to compare the qualitative qPCR results for S1, S2, and S3 samples from Cochabamba, Tarija, and Aiquile cohorts at baseline and between baseline and follow-up time point samples from each treatment group in both clinical trials. Fisher's exact test was used to compare the qPCR sensitivity using two or three replicates, as well as one, two, or three serial samples, and to compare the qPCR positivity between the baseline samples from Cochabamba, Tarija, and Aiquile cohorts, as well as the cumulative therapeutic failure at the end of the 12-month follow-up within each treatment group using one, two, or three serial samples and between BZN arms from both trials. Kruskal-Wallis nonparametric analysis of variance was used to compare the medians of the parasitic loads of quantifiable samples from Cochabamba, Tarija, and Aiquile cohorts at baseline and from each treatment group at baseline and follow-up time points. The Tukey's criterion was used to detect samples with outlier *C_T_* values of IAC (*C_T_* values of >75th percentile plus 1.5× interquartile distance of median *C_T_*) (25). All analyses were performed using SPSS Statistics for Windows V17.0 (SPSS, Chicago, IL).
